# Postoperative Metamorphopsia in Macula-Off Rhegmatogenous Retinal Detachment: Associations with Visual Function, Vision Related Quality of Life, and Optical Coherence Tomography Findings

**DOI:** 10.1371/journal.pone.0120543

**Published:** 2015-04-08

**Authors:** Mathijs A. J. van de Put, Jelle Vehof, Johanna. M. M. Hooymans, Leonoor I. Los

**Affiliations:** 1 Department of Ophthalmology, University Medical Center Groningen, University of Groningen, Groningen, the Netherlands; 2 Department of Twin Research & Genetic Epidemiology, King’s College London, St. Thomas’ Hospital, London, United Kingdom; 3 W.J. Kolff Institute, Graduate School of Medical Sciences, University of Groningen, Groningen, the Netherlands; Justus-Liebig-University Giessen, GERMANY

## Abstract

**Purpose:**

To evaluate postoperative metamorphopsia in macula-off rhegmatogenous retinal detachment (RRD) and its association with visual function, vision related quality of life, and optical coherence tomography (OCT) findings.

**Methods:**

45 patients with primary macula-off RRD were included. At 12 months postoperatively, data on metamorphopsia using sine amsler charts (SAC), best corrected visual acuity (BCVA), letter contrast sensitivity, color vision (saturated and desaturated color confusion indexes), critical print size, reading acuity, the 25-item National Eye Institute Visual Functioning Questionnaire (NEI-VFQ-25), and OCT, were obtained.

**Results:**

Metamorphopsia was present in 39 patients (88.6%), with most of them (n = 35, 77.8%) showing only mild metamorphopsia (SAC score = 1). Patients with metamorphopsia had significantly worse postoperative BCVA (p = 0.02), critical print size (p<0.0005), and reading acuity (p = 0.001) compared to patients without metamorphopsia. Other visual function outcomes and NEI-VFQ-25 overall composite score were all also somewhat lower in patients with metamorphopsia, but this did not reach statistical significance. No association with OCT findings was present.

**Conclusion:**

The prevalence of postoperative metamorphopsia in macula-off RRD patients is high, however, the degree of metamorphopsia is relatively low. When metamorphopsia is present, visual functions seem to be compromised, while vision related quality of life is only mildly affected.

## Introduction

The incidence of rhegmatogenous retinal detachment (RRD) is around 20 cases per 100,000 person-years. Approximately half of the cases present themselves with macula-off RRD.[1] In addition, postoperative recovery of visual acuity, contrast sensitivity, and color vision may be unsatisfactory, even after successful surgery.[2] In macula-off RRD, postoperative metamorphopsia is a highly prevalent problem that occurs in around two thirds of patients.[3–4]

The pathogenesis of postoperative metamorphopsia remains controversial. Most likely, it is caused by disturbances in normal retinal anatomy due to poor orientation of photoreceptors after surgery. Studies on both postoperative metamorphopsia and optical coherence tomography (OCT) have revealed associations between metamorphopsia and the presence of an epiretinal membrane (ERM)[3] and/or subretinal fluid.[3–4] However, OCT findings in patients with postoperative metamorphopsia are often normal.[3–4] In these cases, it has been suggested that metamorphopsia may occur due to microstructural photoreceptor disruption that may be undetected by spectral domain OCT examination.[5]

Although many patients present themselves with postoperative metamorphopsia after macula-off RRD, it remains unknown whether this is associated with the outcomes of other postoperative visual function tests, and vision related quality of life. For instance, previous studies showed that lower visual function (best corrected visual acuity (BCVA), letter contrast sensitivity, and color vision disturbances) after surgery for macula-off RRD is associated with lower vision related quality of life scores.[6–8] The main goals of this study are to determine whether postoperative metamorphopsia in macula-off RRD patients is associated with postoperative visual function (best corrected visual acuity (BCVA), letter contrast sensitivity, color vision disturbances, critical print size, and reading acuity) and with vision related quality of life. In addition, we evaluated whether metamorphopsia is associated with OCT findings.

## Methods

### Study design

We conducted a prospective observational study. The research protocol was approved by the University Medical Center Groningen (UMCG) review board ethics committee, and was carried out in accordance with the tenets of the declaration of Helsinki. All patients had given their written informed consent. The study was registered with the Dutch Trial Register (NTR839). All patients were operated on at the ophthalmology department of the UMCG. The study was carried out over a three-year period (February 1, 2007—February 1, 2010).

### Study population

Patients of 18 years or older, with a first presentation of a unilateral macula-off RRD of 24 hours to 6 weeks duration, who underwent successful reattachment surgery within 24–72 hours after presentation at our hospital, were invited to participate in this study. Patients with a macular detachment of < 24 hours were excluded, because in our current treatment strategy these are scheduled as emergency surgeries and inclusion in the study would lead to treatment delay. Patients with macular detachment of more than 6 weeks duration were excluded, because they are considered rare and yield a worse prognosis.[9] Excluded were patients with bilateral RRD, a history of congenital or acquired pathology with an effect on visual function in one or both eyes, or pathology observed at presentation after their macula-off RRD (i.e. pathology of the cornea, lens, vitreous body, retina (including macula and optic nerve, and scleritis). However, patients with a congenital defect in color vision were not excluded. In addition, patients who had a re-detachment during the follow-up period were excluded from the analysis.

### Preoperative measurements

We acquired the following patients’ characteristics: age, gender, affected eye, and ophthalmic history. The surgical technique, i.e. scleral buckling or trans pars plana vitrectomy surgery (TPPV), was documented. Using standardised protocols, the refractive error and BCVA using the Early Treatment of Diabetic Retinopathy Study (ETDRS) chart were determined for both the affected eye and the fellow control eye. All BCVA measurements were converted to logMAR equivalents of ETDRS acuity for analysis. Light perception or hand movements were coded as logMAR VA of 3.0.

### Postoperative measurements

#### Metamorphopsia

At 12 months postoperatively, all patients were tested for perceived metamorphopsia by showing them a standard Amsler chart, first at the non-operated eye and then at the operated eye. In case of perceived metamorphopsia, the modified sine Amsler charts (Amsler SAC) were used, to assess the degree of metamorphopsia semiquantitatively.[10] The modified sine Amsler charts we used consist of six different modified Amsler charts. In the center of each chart, the straight lines of the regular Amsler chart have been replaced by sinusoid lines with a constant frequency but increasing amplitudes, resulting in representations of different grades of metamorphopsia. The lines of the different SAC are described by:

f(x)=b⋅sinx,

where the amplitude ***b*** increases with 0.25 for every consecutive chart. Scores on the sine Amsler charts ranged from 0 (no metamorphopsia) to 6 (severe metamorphopsia).[10]

#### Visual function

At twelve months postoperatively, we measured BCVA using the ETDRS chart, letter contrast sensitivity using the Pelli Robson chart[11], Farnsworth D-15 saturated, and Lanthoni desaturated color confusion indexes (CCI)[12], critical print size and reading acuity using the Laboratory of Experimental Ophthalmology (LEO) reading chart. Critical print size is defined as the smallest text a patient can read. Reading acuity is the smallest text size a patient can read correctly at his maximum reading speed. All measurements were performed in the affected and fellow control eye.

#### Cataract

At 1, 3, 6, and 12 months postoperatively, a full ophthalmic examination was done, including an assessment of BCVA, and an examination of the fundus. Because of an increased risk of cataract development after trans pars plana vitrectomy, which may influence postoperative measurements, we scored the level of cataract using the lens opacities classification system III (LOCS III) in both eyes at all visits.[13] In case of a visually significant cataract, a cataract extraction was performed before the 12 month measurement. In addition, LOCS gradings were performed in all phakic patients at the final visit.

#### Vision related quality of life

At twelve months postoperatively, patients were requested to self-administer the validated Dutch version of the National Eye Institute Visual Functioning Questionnaire (NEI-VFQ-25) to assess their Vision Related Quality of Life (VR-QOL).[14] This questionnaire has been developed by the research and development corporation (RAND), and by the National Eye Institute (NEI). The NEI-VFQ-25 comprises 25 items that require the patient to assess the influence of visual disability and visual symptoms on generic health domains such as emotional well-being and social functioning, in addition to task-oriented domains related to daily visual functioning.[15–16] Each item is assigned to one of the following twelve subscales: general health, general vision, ocular pain, near activities, distance activities, vision specific social functioning, vision specific mental health, vision specific role difficulties, vision specific dependency, driving, color vision, and peripheral vision. Each subscale consists of a minimum of one and a maximum of four items. We used the standard algorithm to calculate the scale scores. The subscales range from 0 to 100 points, where 100 indicates the highest possible function. The NEI-VFQ-25 overall composite score (OCS) is calculated as the unweighted average response to all items, excluding the question on general health.

#### Optical coherence tomography

At 12 months postoperatively, OCT measurements were performed using the STRATUS 3000 OCT (Carl Zeiss Ophthalmic Systems, Dublin, CA) by a single trained observer (ophthalmic technician) who was only active in collecting, and grading the data independent of the outcomes of the other measurements. The cross hair scan acquisition protocol was used for qualitative examination of the macular region. The presence (yes or no) of intraretinal cystic spaces, subretinal fluid, vitreomacular traction (VMT), and epiretinal membrane (ERM) were assessed. The fast macular scan acquisition protocol was used for quantitative examination of central retinal thickness at the foveal dip, defined as the distance between the internal limiting membrane and the outer surface of the photoreceptor layer. Using the analysis program, a caliper measurement was carried out by placing one caliper on top of the retina, at the location of the foveal dip, and the other caliper on the second high reflective line (retinal pigment epithelium). The acquired value was used to quantify central retinal thickness in three classes based on normative data; atrophic (thickness < 135 μm.), within normal limits (thickness ≥ 135 μm. and ≤ 205 μm.), or hypertrophic (thickness > 205 μm.).[17]

### Statistical Analysis

Data were analyzed using SPSS software package, version 22.0 (Chicago, Illinois, USA). All outcome variables were first tested for normality and transformation to a normal distribution and/or exclusion of outliers was considered. In case of a normal distribution a *t-test* was used to compare the difference of the vision-related outcome measures and postoperative vision related quality of life in patients with and patients without metamorphopsia. In case of a non-normal data distribution, a *Mann-Whitney U-test* was used to compare this difference. We performed the same analyses in the subgroup of patients without OCT disturbances only. We used a Fisher’s exact test to determine if postoperative OCT disturbances were significantly more frequent in patients with versus patients without postoperative metamorphopsia. The significance level of this study was set at a 0.05, two-sided.

## Results

A total of 56 patients gave their written informed consent and were included in the study. Ten patients had one or more re-detachments and one patient deceased during the study period, and these were subsequently excluded. In total, data of 45 patients were analyzed in this study. Table 1 summarizes patient and ocular characteristics and type of surgery.

**Table 1:** Data on patients, operated eyes and surgical procedure.

Characteristics	Mean ± SD / Median (ranges) / n (%)
Number of patients (eyes)	45 (45)
Age (y)	61 ± 10.6
Sex (male/female)	34 (76%) / 11 (24%)
Right eye/left eye	22 (48.9%) / 23 (51.1%)
Preoperative lens status (Phakic / pseudophakic)	29 (64%) / 16 (36%)
Postoperative lens status (Phakic / pseudophakic)	8 (18%) / 37 (82%)
Surgical procedures (scleral buckling/TPPV)	6 (13%) / 39 (87%)
Preoperative BCVA (LogMAR)	3.00 (0.06–3)
Postoperative foveal thickness at 12 months	177 (68–562)
Postoperative metamorphopsia score (SAC) at 12 months	1 (1–6)

SD: Standard deviation, TPPV: Trans pars plana vitrectomy. BCVA: Best corrected visual acuity. SAC: Sine Amsler chart.

A TPPV (n = 39, 86.7%), with (n = 22) or without (n = 17) an encircling band was most frequently chosen as the primary surgical procedure, and in the remaining 6 cases (13.3%) a scleral buckling procedure was used. During the 12 months follow-up period, cataract surgery was performed in 21 of 29 pre-operatively phakic eyes, and the LOCS scores in the remaining 8 phakic eyes did not significantly influence visual function variables (data not shown). During this period, no other visual co-morbidities were diagnosed. Twelve months postoperatively, metamorphopsia was present in 39 operated eyes (88.6%), with most of those (n = 35, 77.8%) showing only mild metamorphopsia (Amsler SAC score = 1). None of the fellow eyes had metamorphopsia.

Critical print size and desaturated CCI turned out to have a normal distribution and were tested using a *t-test*. All other outcome variables (logMAR visual acuity, log letter contrast sensitivity, color vision (saturated and desaturated CCI), critical print size, reading acuity, and NEI-VFQ-25 OCS and subscales) could not be transformed to a normal distribution and were tested using a *Mann-Whitney U-test*. The mean scores of patients with and without metamorphopsia on visual outcomes and quality of life overall composite score 12 months postoperatively are provided in Table 2.

**Table 2:** Postoperative (12 months) visual function and vision related quality of life in macula-off RRD patients with and without metamorphopsia.

	Total group (n = 45)			Patients without OCT abnormalities only (n = 22)		
Visual function	Metamorphopsia (n = 40) Mean (s.e.)	No metamorphopsia (n = 5) Mean (s.e.)	P-value for a difference in groups*	Metamorphopsia (n = 19) Mean (s.e.)	No metamorphopsia (n = 3) Mean (s.e.)	**P-value for a difference in groups** *
BCVA (logMAR)	**0.33 (0.05)**	**0.07 (0.04)**	**0.02**	**0.29 (0.05)**	**0.02 (0.03)**	**0.009**
Letter contrast sensitivity (Log)	1.36 (0.05)	1.54 (0.04)	0.28	1.47 (0.06)	1.53 (0.07)	0.93
Saturated CCI	1.43 (0.08)	1.03 (0.03)	0.07	1.40 (0.12)	1.04 (0.04)	0.41
Desaturated CCI	1.86 (0.10)	1.61 (0.20)	0.37	1.93 (0.14)	1.76 (0.28)	0.64
Critical print size	**0.53 (0.04)**	**1.02 (0.15)**	**<0.0005**	**0.58 (0.06)**	**1.09 (0.26)**	**0.009**
Reading acuity	**0.33 (0.03)**	**0.67 (0.09)**	**0.001**	**0.34 (0.04)**	**0.77 (0.11)**	**0.003**
**Vision related quality of life**						
Overall composite score NEI-VFQ-25	89.5 (1.1)	94.1 (2.3)	0.07	89.8 (1.6)	95.4 (1.6)	0.11

* Desaturated CCI and Critical print size were tested using a t-test, all other variable were tested using a Mann-Whitney U-test. Significant associations are in bold. s.e.: standard error of the mean, BCVA: Best corrected visual acuity CCI: Color confusion index, NEI-VFQ-25: National Eye Institute Visual Functioning Questionnaire-25.

**Fig 1:**
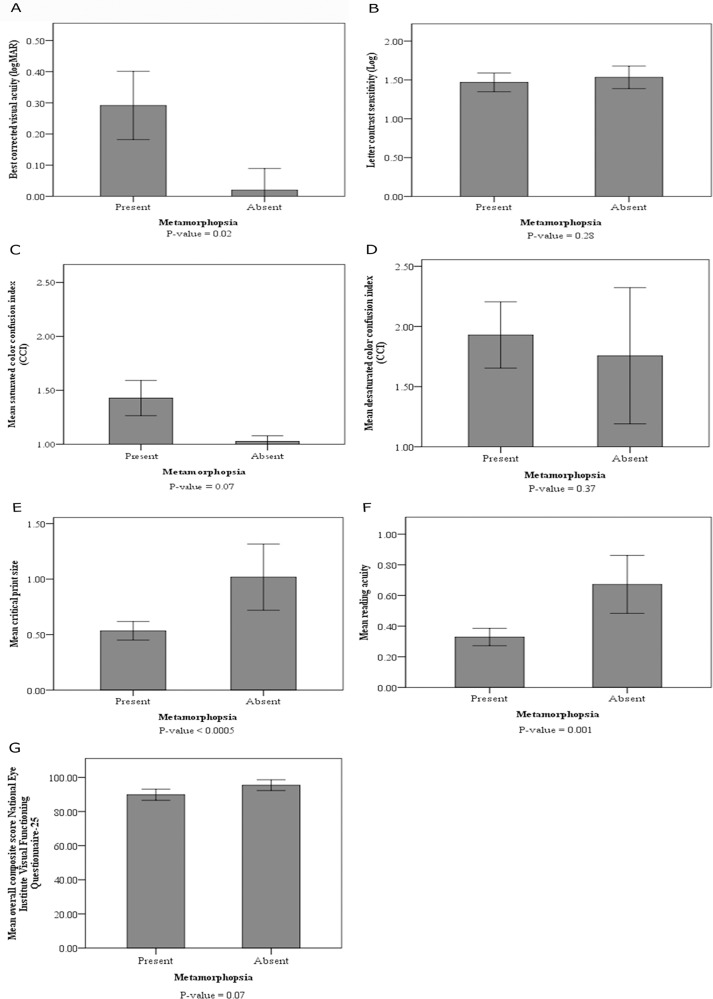
Mean values (± 2 standard errors) of patients with and those without metamorphopsia of a. best corrected visual acuity (logMAR), **b.** letter contrast sensitivity (Log), **c.** saturated color confusion index (CCI), **d.** desaturated color confusion index (CCI), **e.** critical print size, **f.** reading acuity, and **g.** overall composite score National Eye Institute Visual Functioning Questionnaire-25. Differences between groups were tested using an independent t-test (desaturated CCI and critical print size) or using a Mann-Whitney U-test (best corrected visual acuity (logMAR), letter contrast sensitivity (Log), saturated CCI, reading acuity, and overall composite score National Eye Institute Visual Functioning Questionnaire-25) and corresponding p-values are given below the figure.

All visual outcome variables were worse in patients with metamorphopsia compared to patients without metamorphopsia (see Fig 1). BCVA, critical print size, and reading acuity reached statistical significance. As an example, the presence of metamorphopsia was significantly associated with best corrected visual acuity (p = 0.02), leading to a decrease of 0.26 point logMAR in patients with metamorphopsia. There was a trend that vision related quality of life was also lower in patients with metamorphopsia (p = 0.07). Of the NEI-VFQ-25, all 12 subscales except general health showed lower quality of life in the metamorphopsia group (data not shown), but only the subscale near activities reached statistical significance (p = 0.04). In the analysis in the subpopulation of patients without OCT disturbances only (right part of Table 2), we observed a similar effect of postoperative metamorphopsia on postoperative visual function and vision related quality of life. Again, BCVA, critical print size, and reading acuity reached statistical significance.

Postoperative OCT disturbances and postoperative metamorphopsia were both present in 21 patients. In two patients postoperative OCT disturbances were present and metamorphopsia was absent. Postoperative OCT disturbances and postoperative metamorphopsia were both absent in 4 patients. In 18 patients OCT disturbances were absent and metamorphopsia was present. We observed no association between postoperative metamorphopsia and the presence of any postoperative OCT disturbances (p = 0.414). Observed OCT disturbances included: atrophic retina (n = 6, 13.3%), hypertrophic retina (n = 14, 31.1%), intraretinal cysts (n = 4, 8.9%), subretinal fluid (n = 3, 6.7%), and epiretinal membrane (n = 10, 22.2%).

## Discussion

Although the prevalence of postoperative metamorphopsia in this study was high compared to other studies[3–4], the encountered degree of metamorphopsia was small. All visual outcome variables were worse in eyes with metamorphopsia. BCVA, letter contrast sensitivity, critical print size, and reading acuity were significantly worse in operated eyes with metamorphopsia compared to eyes without metamorphopsia. We did not observe a significant difference in vision related quality of life in patients with and patients without metamorphopsia, nor did we observe a significant relation between metamorphopsia and OCT disturbances.

Differences in study design, (in- and exclusion criteria, follow-up, and method to determine metamorphopsia) and patient selection (age, and surgical technique) may be responsible for differences in reported prevalences of postoperative metamorphopsia between our and other studies.[3–4] Important factors such as duration and height of the macular detachment, refractive error, and age probably differed between studies.[3–4] For example, the recovery of metamorphopsia follows a slower time course than the recovery of VA.[18] Therefore, one would expect the prevalence of postoperative metamorphopsia to be higher in studies with shorter follow-up periods. However, in our study with a longer follow-up, we observed a higher prevalence of metamorphopsia than others.[3–4] This may be explained by the fact that other studies included younger patients.[3–4] A younger age at the time of macula-off RRD is a beneficial prognostic factor for the recovery of visual function.[2] Similarly, it may also be a beneficial prognostic factor for the recovery of metamorphopsia. Further, in other studies, TPPV was less often chosen as a surgical technique.[3–4] Perhaps, performing TPPV leads to more disorganization at the level of the photoreceptors, (i.e. unintentional vertical displacement of the retina as was demonstrated by Shiragami et al.) and hence to an increased prevalence of metamorphopsia.[19] Finally, others used a different method to assess metamorphopsia.[3–4] Okamoto used M-CHARTS, and Wang used the regular Amsler grid to assess metamorphopsia.[3–4]

The presence of postoperative metamorphopsia was associated with lower scores on all visual outcome variables and NEI-VFQ-25 OCS. A previous study,[20] observed an improvement in reading ability in patients after TPPV for macular hole and macular pucker (macular pathologies often presenting with metamorphopsia), which seems to be in line with our study. Reading acuity testing will probably be more severely affected by small amounts of metamorphopsia than visual acuity testing. The former task—recognizing words at maximum reading speed—carries a higher rate of difficulty than the latter, in which optotypes can be read separately and reading speed is not an issue. Fortunately for patients, the presence of mild unilateral metamorphopsia after surgery for macula-off RRD does not seem to affect vision related quality of life. In a previous study we observed that other factors (postoperative BCVA, letter contrast sensitivity, and color vision disturbances) are more important in vision related quality of life after macula-off RRD.[6]

One could argue that both visual function loss and metamorphopsia are caused by changes in the organization of the macular area. However, in the analyses in the subpopulation of patients without OCT disturbances, we observed a similar association between the presence of postoperative metamorphopsia and lower scores on all visual outcome variabes and NEI-VFQ-25 OCS. This suggests that in the absence of OCT disturbances the presence of metamorphopsia also directly affects postoperative visual function and NEI-VFQ-25 OCS. However, we cannot exclude the possibility that microstructural changes that are not visible on the OCT we used led to the associations we found.

We, and others observed that OCT disturbances may be absent in patients with postoperative metamorphopsia.[3–4] This may partially be caused by differences in used OCT. It is likely that studies using earlier OCT models report less OCT disturbances than studies using later models, since the latter give a higher resolution. For example, Okamoto used the Cirrus high definition spectral-domain OCT, and observed a higher number of OCT disturbances when compared to our study.[3] A shorter follow-up period is more likely to result in more OCT disturbances as subretinal fluid and retinal cysts tend to disappear in a time span of 6 to 12 months.[4,21] The shorter follow-up period in the study of Wang (who used the lower resolution OCT 2 scanner) may explain their higher number of observed OCT disturbances, when compared to our study.[4] In addition, a longer follow-up period may be related to the inability to observe microstructural changes in the organization of the macular area.

The strengths of our study are the long follow-up period, the standardized measuring methods of metamorphopsia, visual function, vision related quality of life, and the qualitative assessment of OCT. A limitation of our study is the relatively small sample size and the lack of variation in the amount of metamorphopsia that together may lack sufficient power to observe the full relations between variables and outcome measures. The lack of variation in the amount of metamorphopsia may be related to the limited sensitivity of our testing method for metamorphopsia. This may be the reason that the postoperative metamorphopsia score was only around 1. A more sensitive testing method may have resulted in more variation in perceived metamorphopsia.

In conclusion, the prevalence of postoperative metamorphopsia in macula-off RRD patients is high, however, the degree of metamorphopsia is relatively low. When metamorphopsia is present, visual functions seem to be compromised, while quality of life is only mildly affected.

## Supporting Information

S1 Database Database containing patients data. (XLSX)
